# Current status, challenges, and future career pathways of diploma-prepared nurses from the stakeholders’ perspective: a qualitative study

**DOI:** 10.1186/s12912-024-02152-z

**Published:** 2024-08-07

**Authors:** Seema Nasser

**Affiliations:** 1 Department of Nursing, College of Nursing, King Saud bin Abdulaziz University for Health Sciences, Riyadh, Saudi Arabia; 2 King Abdullah International Medical Research Center, Riyadh, Saudi Arabia; 3 Ministry of the National Guard - Health Affairs, Riyadh, Saudi Arabia

**Keywords:** Nursing education, Nursing, Diploma Programs, Curriculum/trends, Curriculum Development, Career pathways.

## Abstract

**Background:**

The global shortage of nurses is a pressing issue affecting healthcare quality and patient outcomes. Nurse turnover is driven by work-related stress, and job dissatisfaction is persistent. In Saudi Arabia, many diploma-prepared nurses need more bridging programs to convert their diplomas into bachelor’s degrees. Educational and organizational issues can limit the provision of quality nursing care. Differences in educational preparation influence nurses’ interpretations of patient safety and their roles within healthcare systems. Addressing the need for more policies and regulations regarding nurse turnover and the retention of diploma-prepared nurses is crucial. Thus, a comprehensive exploration of barriers and incentives for diploma-prepared nurses to complete their Bachelor of Science in Nursing (BSN) can lead to transformative institutional strategies, such as tuition compensation and clinical-academic collaborations. This study aims to fill this gap by understanding the current challenges, future trends, and solutions from stakeholders’ perspectives and developing tailored career pathways for diploma-prepared Nurses from the stakeholders’ perspective. Thus, it contributes to policy development and improved healthcare delivery and fosters a promising future for healthcare.

**Methods:**

This qualitative study employed a thematic analysis and grounded theory methodology as we delved into stakeholders’ perspectives to generate a substantive framework for overcoming obstacles and cultivating tailored career pathways for diploma-prepared nurses. A purposive sampling technique was used to choose participants, ensuring their rich, relevant, and diverse information based on their expertise, experience, and ability to provide valuable insights. Data were collected using one-on-one semi-structured questions for in-depth interviews.

**Results:**

Our findings revealed key concepts that were evident in the data. These concepts formed three main themes and several subthemes essential to understanding the *current status of*,* challenges faced by*,* and career pathways* for diploma-prepared nurses. The three main themes have emerged, and core categories have emerged under each theme accordingly. The results generated a practical framework, offering tangible solutions to overcome challenges and develop career pathways for diploma-prepared nurses.

**Conclusions:**

The findings significantly affect policy development and healthcare delivery improvement. This suggests the need for policies that support diploma-prepared nurses in completing their BSN and the development of tailored career pathways that align with their educational background and career goals and the Kingdom’s 2030 Vision.

**Supplementary Information:**

The online version contains supplementary material available at 10.1186/s12912-024-02152-z.

## Background

The worldwide shortage of nurses has reached critical levels, exacerbating the demand for nursing services [1, 2] and prompting an urgent need for proficiently trained registered nurses. This shortage is not just a statistic but a pressing issue affecting healthcare quality and patient outcomes. However, nurse turnover remains a persistent issue, fuelled by work-related stress and job dissatisfaction, thus emphasizing the imperative of retaining nurses in long-term positions [3]. The current nursing workforce in Saudi Arabia includes many diploma-prepared nurses, with some not employed due to the lack of bridging programs to convert their diplomas into bachelor’s degrees [4]. Educational and organizational issues related to diploma-prepared nurses can limit the provision of quality, advanced nursing care [4]. Highly educated nurses tend to be better prepared for managerial roles, more engaged in research, and more proficient at implementing best-practice guidelines [5, 6]. Studies indicate that differences in educational preparation influence nurses’ interpretations of patient safety and their roles within healthcare systems [6].

It is essential to address the need for more policies and regulations regarding nurse turnover and the retention of diploma-prepared nurses [4]. Therefore, a comprehensive exploration of the barriers and incentives for diploma-prepared nurses to complete their BSN can lead to effective institutional strategies, such as tuition reimbursement and academic collaborations [7]. Studies have identified the primary themes of sacrifices, barriers/challenges, incentives/support, value, initiation, and pressure [7]. By enhancing accessibility to educational programs and financial support, these barriers can be overcome, ensuring the completion of BSN degrees [7]. Research indicates diverging labor market outcomes for associate degree-prepared nurses in the United States of America (USA), with many transitioning from hospital employment to longer-term settings [8, 9]. Existing literature underscores the benefits of a baccalaureate degree for nurses and patients and calls for policies supporting diploma-prepared nurses in pursuing further education [10]. Employers can influence educational choices by offering support and flexibility [10]. This connection highlights the need for research addressing this specific issue.

The significance of exploring the underexplored aspects lies in the potential to enhance patient outcomes and the quality of care by retaining and advancing diploma-prepared nurses within the profession. Thus, attending to the nursing shortage and improving nursing education is critical to enhancing patient care quality in Saudi Arabia, aligning with the Kingdom’s 2030 Vision [11]. Nursing leaders, policymakers, and stakeholders’ roles in the healthcare industry are crucial, with the necessity of active involvement and support in implementing the proposed solutions, which will lead to significant transformations in the nursing workforce and healthcare delivery in Saudi Arabia.

Previous studies have delved into the impact and determinants of nurse turnover [3, 12–14], but few have specifically addressed the career pathways of diploma-prepared nurses, particularly in Saudi Arabia. Although international bridging programs exist, many diploma or associate-degree-prepared nurses in the USA have not pursued bachelor’s degrees due to financial constraints, inflexible work schedules, and family commitments [15]. Continuing education and increased educational credentials in nursing offer benefits such as a higher likelihood of promotion, increased incomes, and improved patient outcomes [16–18]. While past studies have described the experiences of diploma-prepared nurses, there needs to be more evidence of their career paths and retention in Saudi Arabia. This study aims to fill this gap by understanding the current challenges, future trends, and solutions from stakeholders’ perspectives, ultimately contributing to policy development and improved healthcare delivery. This gap is crucial as it hinders understanding how to retain these nurses effectively in the workforce. This study uniquely focuses on generating tailored career pathways, filling a critical gap in the existing literature, and offering innovative solutions to address nurse retention issues, specifically for diploma-prepared nurses in Saudi Arabia.

Therefore, the objectives of this study were to determine the current status of diploma-prepared nurses from stakeholders’ perspectives, identify their challenges, and develop tailored career pathways for them. It aimed to create a framework to overcome challenges and support diploma-prepared nurses in advancing their careers. The research findings hold significant potential for diploma-prepared nurses, offering insights that can help them overcome their challenges and advance in their careers. These solutions, when implemented, can lead to a more robust nursing workforce, improve patient outcomes, and enhance healthcare delivery in Saudi Arabia. Furthermore, these findings contribute to the field by addressing the urgent need for strategies to retain diploma-prepared nurses and improve nursing education aligned with the Kingdom’s 2030 Vision [11], with the ultimate goal of dedication and commitment to contributing directly to the healthcare industry.

## Methods

### Research procedure

This qualitative study utilized an empirically derived substantive framework to overcome challenges and develop tailored career pathways for diploma-prepared nurses from the stakeholders’ perspective. A thoroughly chosen purposive sampling technique was used in this qualitative research to select participants who can provide relevant and diverse data to the research questions, which aligned with the study’s objectives layered by flexibility and adjustments as the study progressed to include additional participants who may provide new insights. Stakeholders from central Saudi Arabia were chosen based on their position and experience to provide valuable insights into the challenges and potential solutions for diploma-prepared nurses. Using purposive sampling ensured that the study captured a comprehensive understanding of the issues from various perspectives, enhancing the depth and relevance of the findings.

We recruited participants until saturation was achieved, including representatives from the Saudi Commission for Health Specialties, representatives from the Professional Nursing Council, a representative from the Scientific Nursing Council, the General Director of Nursing at the Ministry of Health, a consultant from the Health Academy for Saudi Commission for Health Specialties, and representatives from the Riyadh First Health Cluster initiative: Executive Director of Nursing in King Saud Medical City and the leader of the nursing sector in the cluster initiative. Therefore, the inclusion criteria encompassed stakeholders responsible for policymaking and decision-making in the nursing profession, from the Ministry of Health, Saudi Commission for Health Specialties, Health Academy for Saudi Commission for Health Specialties, and Riyadh First Health Cluster initiative. Thus, Eligible subjects were identified in collaboration with the coordinator of Riyadh First Health Cluster (Supplementary material 1: Characteristics of the study Participants).

### Research instrument

The data collection process was designed to ensure comprehensive and reliable results using one-on-one semi-structured questions for the in-depth interview, which lasted one hour with probing questions to help gather data for this research (Supplementary material 2). The questions were tailored according to each identified stakeholder’s job descriptions, roles, and responsibilities. All questions were in Arabic, except for the questions for the representative consultant of the Health Academy for Saudi Commission for Health Specialties, which was in English. Interviews were conducted at a place and time agreed upon by the participant and interviewer.

### Data management and analysis plan

Thematic analysis process was conducted rigorously to ensure the validity and credibility of the study’s conclusions. Furthermore, the grounded theory methodology was employed to produce a detailed description and thematic analysis of the challenges faced by and the future of diploma-prepared nurses. Grounded theory is beneficial for focusing on research areas with little information [19, 20]. The goal of data analysis was to compare data to discover potential core categories, which would improve the understanding of the *current status of*,* challenges faced by*,* and career pathways* for diploma-prepared nurses. Data collection and analysis co-occurred, as data were transcribed verbatim; word-by-word and line-by-line analyses were conducted with constant comparison techniques to derivate substantive concepts [19, 20]. Through constant comparison, the researcher grouped data representing similar facets into similar categories and developed themes and concepts accordingly.

The researcher used the memoing technique to aid conceptualization and theory building, which helped discover recurring core categories in the data [19, 20]. The core categories were identified, and the researcher and an expert researcher in qualitative research began consensus coding to effectively ensure a high level of rigor as the researcher and the expert coded the same transcripts and compared results on a one-to-one basis. Then, we proceeded with selective coding, which refers to limiting coding by theoretical sampling to only data related to the core categories. When theoretical coding was saturated, the researcher, with the expert, sorted memos to develop hypotheses about concepts and help ensure the parsimony of the substantive framework. The linkage between concepts was verified to generate a substantive framework. The researcher reviewed and compared the data to other data and codes already developed.

## Results

The thematic analysis process applied to the transcribed interviews elicited key concepts that were evident in the data. The key concepts formed three main themes and several subthemes that are essential to understanding the current status of, challenges faced by, and career pathways for diploma-prepared nurses (Supplementary material 3).

The three main themes are “**current status**,” “**challenges**,” and “**career pathways**,” and the results generated a framework to overcome challenges and develop career pathways for diploma-prepared nurses. Core categories have emerged under each theme, which will be discussed in the following section.

***Current Status***: The first theme is the current status of diploma-prepared nurses, which have been divided into the following three core categories: career aspirations, practice competently, and education and training standing.

### Career aspirations

This is the first category of the emergent framework. Participants described “career aspiration” as an understanding of the “big picture” of what was going on with diploma-prepared nurses throughout their careers. Nurses can fall under any one of the following categories: ***willing to bridge***, ***in-between careers***, or ***far away from their career***. Stakeholders spoke about the necessity of understanding nurses’ career aspirations to deal with the current situation and better address the needs of the healthcare system. For each, there was a link between the developed, tailored career pathways. One participant emphasized their perspective on career standing:

…*Here’s the three sides. Some of them want to bridge, and some are a little far away. Some are between one and the other. It all depends on the circumstances they have. They tend to be on the first side or younger and unmarried, especially the ladies*.

### Practice competently

This is the second core category. It describes diploma-prepared nurses who practice competently through experience and have an authoritative manner as founders and leaders. They are competent and assigned supervisor and head nurse roles, thus they are accounted for as leaders of the nursing profession. They form the foundation of nursing practice as advanced experts, yet they are labeled as technicians. Several participants stated.

*They are competent, highly experienced 10–15 years when compared to BSNs… in my opinion they are the leaders of nursing practice*.

*They were always given authority… I have respect for them since they are the pioneers and the pillars….*

*I must say that we really exceed the percentage of the International Standards of Diploma prepared and associate degree as called in the USA.*

*They are the Founders of Key performance indicators (KPIs) and developer hospitals. They are founders of policy and procedure as they also lead to the accreditation of many hospitals.*

### Education and training standings

It is explained as a need for more theoretical education. The nurses in this category are not targeted in training and thus need more overall training. As participants explained.

*They are trained well… but they don’t have the background to compare between inotrope vs. vasopressor… prevention of complications and understanding anatomy and physiology… so on.*

*…From a knowledge perspective, they don’t differentiate between pharmacodynamics or pharmacokinetics unless someone is genius and smart and reading more. Otherwise, I’m still in doubt.*

*they are never targeted all continuous educations courses… Only BSN and above… although they always show up and ask to join…*.

***Challenge and Consequences***: The second theme of the framework encompasses six main core categories: no existing scope of practice, unfair in their right to education, no existing regulations for part-time enrolment, suffer financial burden, blame the Ministry of Health for their limited opportunities, and suffer from discrimination.

### No existing scope of practice

There was no existing scope of practice at the time the study was conducted, and the interviews were taken, but there were plans for such initiatives in the future. However, this category spoke to challenges with practice, classification, qualifications, and more so because diploma-prepared nurses are constantly threatened by bachelor-prepared nurses, as addressed by participants. Future regulations might lead them to stop practicing if their issues are not addressed. Nevertheless, there needs to be consideration for their years of experience as functioning registered nurses and in different managerial roles. Participants mentioned the following in their interview.

*The main problem is there is* ***no existing scope of practice****. We are currently working on it … Once finalized they will not be able to practice… and they are threatened by future regulation (won’t practice)*.

*Many challenges with* ***classification and qualifications*** *as they will always be technicians*.

*It saddens me deeply that there is* ***no consideration for no. Years*** *of experience*.

### Unfair in their right to education

The second major category for challenges is explained as the reality of no existing bridging programs, and international bridging programs with no practical training and a lack of further educational opportunities only aimed at those who already have a BSN. This has led these diploma-prepared nurses to opt out of continuing their education due to challenges with their qualifications. Due to the lack of theoretical background provided by diplomas, nurses are willing to continue their education and enrol in bridging programs. In terms of overall training, they are targeted as something other than technicians. Courses are always targeted at those who have a BSN or higher qualifications. Diploma-prepared nurses strive for and desire development but need more training opportunities. As most participants agreed.

*They are never targeted all continuous educations courses… Only BSN and above… although they always show up and ask to join….*

### ***Lack of development and lack of accredited governmental programs***

Diploma-prepared nurses are never targeted for courses, and there are no existing regulations for part-time educational enrolment. The available programs always necessitated full-time enrolment. As explained by participants:

…*considering that they are experienced, and they did lead to the accreditation of many hospitals… they have the right to further education.*

### Suffer financial burden

This encompasses one main idea. Existing private programs are not equivalent to financial capacity to manage their own financial affairs with no part-time enrolment limitations, as one participant highlighted.

*…The challenge that the government programs stopped… and it is not clear why… and the existing private programs …not equivalent to financial capacity … and that hinders them from continuing….*

### Blame the Ministry of Health for their limited opportunities

This category speaks to the consequences of challenges. It was an evident category; challenges have affected nurses’ confidence as they weren’t considered a priority in the workforce due to their qualifications. This was highlighted by all stakeholders. They complain and blame the Ministry of Health for their limited opportunities, leading to the sixth category of challenges.

### Suffering from discrimination

This is another clear core category of the consequences of challenges. Participants delineated this in their responses.

*…We really believe that they suffer and not prioritized as others with BSN and master because of their qualification. And they are simply not a priority put last, and the system discriminates them unintentionally because of qualification*.

*…they really complain and maybe more so blame the Ministry of Health for not giving them chances, options and….*

***Tailored career pathways***: The last theme consists of suggested solutions divided into the following five core categories: specific primary goals for career pathways, collaborations between stakeholders, regulations and policymaker initiatives, characteristics of recommended programs, and expected outcomes of the career pathways. Though interviewed stakeholders were referring to the current status of the nurses, there was a clear link in their discussions to the three categories of ***willing to bridge***,*** in-between***, and ***far away***. It was delineated that those far away have no option but to change careers and eventually repurpose, but the stakeholders emphasized the need to focus on the two first types.

### Specific primary goals for career pathway

This is the first category stressed for the need to target diploma-prepared nurses to address the needs of society and the nursing workforce. There is a need to increase the % of highly qualified nurses. For example, participants stated.

*….to keep up with the directions of the Ministry of Health…We need to focus on Optimal investment of human resources*.

*…the goal is to increase the % of highly qualified nurses by energizing the scientific/theoretical aspect*.

### Collaborations between stakeholders

A major core category for the tailored career pathway theme entails collaboration with hospitals and strategic partnerships between the Ministry of Health, Ministry of Education, SCSFH, universities, hospitals, and the SCHFS to aid classifications and qualifications. Furthermore, core collaborations with universities are required to adopt academically accredited programs. Such partnerships are for the sustainability of the process rather than inspection, and they thus can enhance the quality of the program through the continuity of stakeholders’ partnerships. Besides increasing the opportunities for government funding and support, this is linked to the quality of the programs. Regulations will also assist in continuous and sustainable quality. Additionally, the participants emphasized that this can build collaborations with the labor market and all stakeholders. Thus, there is greater emphasis on agile development and collaborative enforcement with training entities. As several participants mentioned.

…***Strategic partnership ****between Ministry of Health, Ministry of Education, Saudi Commission for Health Specialties, hospitals, and universities…. Will be the key to successful implementation …. Most important…*.

***Adoption ****by SCHFS to aid classifications and qualifications while Universities adopt the academically accredited program*.

### Regulations and policymakers’ initiatives

This involves development towards the standardization of practice and the need for professional career pathways after developing scopes of practice with clearly defined ethical frameworks. Additionally, it includes the unification of the scope of practice in Saudi Arabia with new legislation for part-time and full-time educational requirements and mandatory guidelines for practice with targeted solutions to overcome professional exposure. Thus, substitutions could be made with competent Saudi-qualified nurses by adjusting the qualification requirements.

…*There are many projects that we are working on towards standardization of practice and scope of practice in Saudi Arabia needs to be same all around not each hospital will have their own thing….*

…*There is a huge demand for a professional career pathway that is guided by the codes of practice, ethical frameworks, and mandatory guidelines for practice….*

…*You see the difficulty in continuing education is because of the existing policies of full-time enrolment needs to change….*

*We need to think of solutions in order to attend to the existing situation of professional exposure… through substitution with Saudi competent qualified nurses by adjusting qualification.*

### Characteristics of suggested programs

A unique program with theoretical and specialty tracks necessitates blending accredited bridging clinical programs. Yet, tailored programs are required to factor in experience. These should not require full-time enrolment and allow part-time enrolment as well. Different tracks will serve different specializations, such as clinical, administrative, and educational, which will thus enable nurses to climb the career ladder. These programs should be tailored to be flexible, non-linear, not strict, and yet cautious about career pathways. Accredited, blended bridging clinical programs with collaborations between universities and workplaces will allow for on-the-job training.

*…. some of them have 10–15 years… we can’t start from the beginning as if they are freshman 1st year college … what about their years of experience….*

…. *I have nurses who have ten years experience in adult ICU. Why should I take them to peds and maternity… This incorrect utilization of recourses and experiences.*

*We need flexible, linear, not strict, and cautious programs….*

*…collaborations between universities and workplace is a must with on-job training.*

### Expected outcome of the career pathways

Stakeholders have a vision about expected outcomes that catalyse these programs to enhance clinical reasoning and decision-making while empowering diploma-prepared nurses and providing authority and opportunities. This will lead to a clear understanding of diploma-prepared nurses’ needs, emphasizing empowering subspecialties, enhancing diploma-prepared nurses’ clinical performance, and providing tailored specialty certifications.

…*These programs will enhance clinical reasoning, and they enhance clinical decision making… Enhance their clinical performance… Patients will benefit….*

*…it will empower and give authority.*

*Empower subspecialties and lead to better nursing… Providing tailored specialty certifications will retain them where they have always been, and it will qualify them more….*

In conclusion, themes and core categories have been illustrated in the provided framework in (Supplementary Material 4: Framework for Current Status, Challenges and Future Career Pathways of Diploma-prepared-Nurses from the Stakeholders’ Perspective & Solutions: tailored Career Pathways for Diploma-prepared Nurses from the Stakeholders’ Perspective).

### Explanation of the Generated Framework and Tailored Career Pathways:

The generated framework has indicated that diploma-prepared nurses are divided into three groups: those willing to bridge, those in between, and those entirely far away. Specifically, those willing to branch illustrate current status situations regarding educational training and their practice barriers. In contrast, these current statuses impose specific challenges that pertain to academic, financial, and practical difficulties. The consequences of these challenges have led to the suggestion of several solutions from the stakeholders’ perspectives: virtual programs, continuous education, and clinical blended bridging (Supplementary Material 4).

More specifically, A unique program for Tailored Career Pathways that necessitates several aspects: (1) Accredited, blended bridging academic-clinical programs that are tailored to factor in experience, thus fostering collaborations between universities and workplaces will allow for on-the-job training. (2) Theoretically focused specialty tracks tailored to factor in their previous experiences but tend to need more theoretical necessary background tailored according to specialization (3) It should not require full-time enrolment but also allow part-time enrolment and on-the-job training facilitated by the intended clinical-academic collaboration. (4) Distinct tracks will serve different career promotion pathways, such as clinical, administrative, and educational, which will thus enable nurses to climb the career ladder. (5) tailored to be flexible, non-linear, not strict, and yet promote continuous development to facilitate career pathways (Supplementary Material 4).

These solutions, which require collaboration with the need for legislation and policymaking and well-defined and tailored characteristics of the intended programs, highlight the importance of each stakeholder’s role and contribution, making them feel included and valued. Thus, collaborations need to include all the stakeholders from the Ministry of Health, Health care institutions, hospitals, the Saudi Commission for Health Sciences, and the Ministry of Education, including universities, and the inclusion of health clusters, which is necessary, as indicated. Conversely, the suggestion of repurposing through the Academy of Saudi Commission for Health Sciences will be specifically tailored to those far away. As shown in the framework, these solutions will lead to the expected desired outcomes that will enhance clinical reasoning and decision-making while empowering diploma-prepared Nurses and providing authorities and opportunities through tailored specialty certifications.

## Discussion

There is a need to institute policies to support and encourage nurses to pursue BSN degrees [10]. The stakeholders’ sensitivity to the topic and involvement in the study are promising. As addressed in previous studies, the sensitivity of policymakers and stakeholders to factors that act as facilitators and barriers to pursuing higher degrees is essential [10]. Thus, as addressed in our framework and coinciding with similar studies, increasing the accessibility to BSN bridging programs is crucial. In addition, employers can also play a vital role in educational choices by providing nurses with more significant support and flexibility [10]. If implemented, our framework will lead to a paradigm shift because it encompasses the solution and starts with defining the current status and the challenges nurses face. Furthermore, it agrees with other researchers who claim that attending to this problem necessitates a shift in workplace dynamics [21].

Earning a baccalaureate education is beneficial for both diploma-prepared nurses and their patients, where evidence like that of studies from American Nurses confirms that many nurses with diplomas or associate degrees did not pursue a bachelor’s degree because of several challenges, including financial constraints, issues with work flexibility, and family commitments [15]. Compelling evidence indicates that exploring the challenges and support of diploma-prepared nurses can lead to implementing institutional strategies [7]. Like our results, but from the nurses’ perspectives, researchers identified the primary themes: sacrifices, barriers/challenges, incentives/support, value, how to begin, and pressure, such as tuition reimbursement and academic collaboration. Similar to work, previous research indicated that nurses enrolled in educational programs while actively working encounter many challenges and obstacles [22]. Thus, as identified in our framework, their recommendations to overcome these barriers and ensure completion of BSN programs emphasized the need for institutional strategies, such as better accessibility to educational programs and financial support aided by collaboration between hospitals and academic institutions [7].

Similar to our study, recent evidence from the USA demonstrates that many associate degree programs, equivalent to diploma-prepared nurses in Saudi Arabia, are experiencing diverging labor market outcomes. There is a drop in the number of nurses who opt for hospital employment, and many shift to more long-term settings [8, 9]. This speaks to the importance of retaining diploma-prepared nurses before they become the *group far away* from our study. This qualitative study thus helped delineate the status of and challenges nurses face. Then, it stemmed into creating innovative, flexible solutions for staff who wish to further their careers in nursing using the holistic Framework for Current Status, Challenges and Consequences, and Future Career Pathways of Diploma-Prepared Nurses from the Stakeholders’ Perspective. As stressed earlier, there is a need to institute policies that support and encourage nurses to pursue BSN degrees and continue their education [10], thus attending to the first two groups identified in our framework (*in between* and *far away*).

The result of this study is consistent with a recent systematic review, which first delineates that a clear career path still needs to be improved, particularly for diploma-prepared nurses. Yet, nurses who undergo professional on-the-job training may be qualified for promotions or significant career advancements [23]. Thus, the outcomes of this study, along with the recent systematic review, will aid policymakers in the development of on-the-job accredited, blended bridging clinical training programs with collaborations between universities and workplaces to improve nurses’ expertise in a range of specializations and prevention of professional leakage to different career pathways.

Our framework, aligned with the Kingdom 2030 Vision, aims to address nursing shortages, underdeveloped nursing education, and an undistinguishable scope of practice (at the time of the study). With the 2030 Vision in mind, many opportunities for social and economic transformation will be needed to resolve and attend to these challenges. However, in 2023, “The Scope of Nursing and Midwifery Practice” was published by the Saudi Commission for Health Specialities & Nursing and Midwifery, which revealed extensive work in identifying and defining the eight domains of the scope of practice for the nurse technician and nurse specialist, and standards and competencies were set accordingly for each domain [24]. Nevertheless, it entailed some considerations and limitations for the nurse technician/diploma-prepared nurses. Although a recent study before the publication of The Scope of Nursing and Midwifery Practice showed various variations in nursing practice and a lack of internal regulations, the results revealed it did not affect nursing duties precisely [25]. However, the recently published Scope of Nursing and Midwifery Practice will affect diploma-prepared nurses [24], leading to specific implications and limitations of practice for diploma-prepared nurses practice and duties. Thus, this emphasizes the importance of the study’s findings, the implementation of the framework, and the development of tailored career pathways for diploma-prepared nurses.

## Implications, limitations, and future research recommendations

Our study findings are distinguished by its innovative stakeholder-centric approach, which carries substantial significance in its implications and direct and indirect impacts on the nursing career. The interactive self-transcribing of the data (active transcribing) ensured the researcher’s profound immersion in the data, providing crucial insights and implications for diploma-prepared nurses. The most notable is developing a framework that could be empirically tested to steer the nursing profession and the implementation of the suggested academic-clinical partnership-tailored career pathways for diploma-prepared nurses, with derived characteristics from the stakeholders’ perspective. The findings of this study will be of immense value to policymakers, hospital administration, diploma-prepared nurses, and the community.

Hence, implementing this framework is essential and holds immense potential, particularly in the 2030 Vision Realization Program context [26]. Specifically, when prioritizing the retention of diploma-prepared nurses, we can make significant strides toward the program’s objectives, especially those related to transforming the health sector and enhancing human capability [26]. Furthermore, enhancing the quality and efficiency of health services will improve patient outcomes and overall healthcare delivery. The suggested characteristics of the academic-clinical partnership-tailored career pathways will facilitate maintaining standards and fostering mastery and nursing discipline for experienced and highly skilled diploma-prepared nurses. This enhances the nursing profession and promotes growth in professional career pathways.

Furthermore, research supports the implication that policymakers should develop blended clinical training programs and that on-the-job training can qualify nurses for promotions and career advancements [23]. However, an important aspect is aligning educational outputs with labor market needs to ensure that the preparation of diploma-prepared nurses will be adequately prepared to meet the demands of the evolving healthcare landscape. Investing in retaining diploma-prepared nurses by implementing the implication mentioned earlier will enhance employability and strengthen the healthcare system’s resilience and sustainability. The suggested implications of implementing this framework and career pathways are comprehensive, encompassing improvements in healthcare quality, nursing workforce development, and ensuring educational alignment with the changing health sector.

While the proposed framework for retaining diploma-prepared nurses offers promising solutions, it is crucial to acknowledge its limitations and identify areas for future research to enhance its effectiveness further. One limitation could be the resource constraints faced by healthcare institutions, particularly in implementing comprehensive retention strategies. Another limitation is the need for more interviews with the Ministry of Education representatives only to ensure and establish academic-clinical partnerships.

Another minor limitation is that some information about the scope of practice under the scientific and professional counsel from the Saudi Commission for Health Specialties was classified and thus could not be addressed during the interviews and at the time of the study. Yet, after the analysis of the results, the newly released Scope of Nursing and Midwifery Practice [24] was reviewed, and it has shed light on crucial considerations and limitations in the practice and responsibilities of nurse technicians/ diploma-prepared nurses. It is critical to recognize that while the absence of internal regulations did not previously affect nursing duties [25], it is now anticipated to have specific implications for diploma-prepared nurses, further emphasizing the importance of the study regardless of unclassified information at the time of interview.

Further research should investigate the factors influencing nurse retention to explore tailored approaches to address diverse workforce needs and the cost-effectiveness of implementing the suggested framework to promote the sustainability of these implications. Furthermore, future longitudinal studies are warranted to track diploma-prepared nurses’ career trajectories and evaluate the consequences of implementation and evaluation of the suggested tailored career-pathway programs’ adequacy and sustainability.

## Conclusion

The results of our research, guided by the stakeholders’ perspective, have generated an empirically derived, substantive framework to overcome the challenges of diploma-prepared nurses and develop academic-clinical partnership-tailored career pathways. These findings not only shed light on the complex challenges faced but also offer innovative solutions, paving the way for improved retention initiatives and the development of comprehensive policies. Moreover, it holds promise in enhancing healthcare delivery by fostering a conducive environment for diploma-prepared nurses, ultimately ensuring better patient outcomes in Saudi Arabia. While the proposed framework provides a solid foundation for addressing the retention of diploma-prepared nurses, it is crucial to underscore the necessity of ongoing research. This research is vital in achieving the goals of the 2030 Vision Realization Program [26] through this continuous improvement to foster a robust and resilient healthcare system.

### Electronic supplementary material

Below is the link to the electronic supplementary material.

Supplementary Material 1

Supplementary Material 2

Supplementary Material 3

Supplementary Material 4

## Data Availability

The data collected during the interviews form the basis for this study’s findings. They are not publicly available because ethical approval was obtained from King Abdullah International Medical Research Center. Accordingly, only the research team involved in the study can access them to protect study participant privacy. The retention period is five years from the publication date. Supporting documents are available to the reviewer upon written request sent to the corresponding author.
