# Supplementary material for: Current status, challenges, and future career pathways of diploma-prepared nurses from the stakeholders’ perspective: a qualitative study
Source: BMC Nurs. 2024 Aug 7;23:542. doi: 10.1186/s12912-024-02152-z (PMC11304612; doi:10.1186/s12912-024-02152-z)
Supplement: Supplementary file 1 — Supplementary Material 1 [file 12912_2024_2152_MOESM1_ESM.docx]

**Supplementary material 1: Characteristics of the study Participants**

| **Recruitment site** | **N** | **Position** |
| --- | --- | --- |
| **Ministry of Health** | **2** | **-General Director of Nursing Affairs**  **-Director of Human Resources** |
| **Saudi Commission for Health Specialties** | **2** | **-Chairman of the Professional Council**  **-Chairman of the Scientific Council** |
| **Health Academy of Saudi Commission for Health Specialties** | **1** | **-Chairman of the Health Academy** |
| **Riyahd Health Cluster One** | **2** | **-Director of academic affairs**  **-Nursing lead in the Academic Affairs** |
