# Supplementary material for: Current status, challenges, and future career pathways of diploma-prepared nurses from the stakeholders’ perspective: a qualitative study
Source: BMC Nurs. 2024 Aug 7;23:542. doi: 10.1186/s12912-024-02152-z (PMC11304612; doi:10.1186/s12912-024-02152-z)
Supplement: Supplementary file 2 — Supplementary Material 2 [file 12912_2024_2152_MOESM2_ESM.docx]

**Supplementary material 2: Semi-structured questions for the in-depth interview**

1. What is the current status of diploma-prepared nurses?
2. What is the career path of a diploma-prepared nurse?
3. What are the problems and concerns they face?
4. What are the challenges they face?
5. What are the solutions proposed to address the challenges?
6. Are there new regulations or suggestions for diploma-prepared nurses?
7. What is the strategic plan for professional development for diploma-prepared nurses? What are the goals and objectives?
8. Is there an executive plan, and who is responsible for following it?
9. What performance indicators are used to verify the arrival of goals or targets in the executive plan?
10. Do you have any additional comments on this subject?
