# Supplementary material for: Current status, challenges, and future career pathways of diploma-prepared nurses from the stakeholders’ perspective: a qualitative study
Source: BMC Nurs. 2024 Aug 7;23:542. doi: 10.1186/s12912-024-02152-z (PMC11304612; doi:10.1186/s12912-024-02152-z)
Supplement: Supplementary file 3 — Supplementary Material 3 [file 12912_2024_2152_MOESM3_ESM.docx]

**Supplementary material 3: Themes derived from the analysis of the interview.**
