# Supplementary material for: Current status, challenges, and future career pathways of diploma-prepared nurses from the stakeholders’ perspective: a qualitative study
Source: BMC Nurs. 2024 Aug 7;23:542. doi: 10.1186/s12912-024-02152-z (PMC11304612; doi:10.1186/s12912-024-02152-z)
Supplement: Supplementary file 4 — Supplementary Material 4 [file 12912_2024_2152_MOESM4_ESM.docx]

**Supplementary material 4: Framework for Current Status, Challenges and Future Career Pathways of Diploma-prepared-Nurses from the Stakeholders’ Perspective & Solutions: tailored Career Pathways for Diploma-prepared Nurses from the Stakeholders’ Perspective**
